# EDTA-Assisted Sonochemical Synthesis of Polymorphic Bismuth Ferrites: Structural and Photocatalytic Characterization

**DOI:** 10.1021/acsomega.5c05699

**Published:** 2025-08-27

**Authors:** Nivaldo Freire de Andrade Neto, Joyce Marina Paiva da Silva, João Marcelo Soares da Cunha, Marcio Daldin Teodoro, Marcio Assolin Corrêa, Mauricio R. D. Bomio, Fabiana Villela da Motta

**Affiliations:** † LSQM—Laboratory of Chemical Synthesis of Materials—Department of Materials Engineering, Federal University of Rio Grande do Norte—UFRN, P.O. Box 1524, 59078-970 Natal, RN, Brazil; ‡ Physics Department, 28123 Federal University of Rio Grande do Norte, 59078-900 Natal, RN, Brazil; § Department of Physics, Federal University of São Carlos, 13565-905 São Carlos, SP, Brazil

## Abstract

This study investigated the sonochemical synthesis of bismuth ferrite (BFO) systems, focusing on how varying EDTA concentrations influence crystalline phases and photocatalytic performance. X-ray diffraction (XRD) analysis showed that 33EDTA uniquely formed single-phase BiFeO_3_, while other EDTA concentrations resulted in secondary phases like Bi_25_FeO_40_ and Bi_2_Fe_4_O_9_. EDTA concentration was crucial for phase control, with higher concentrations favoring the Bi_2_Fe_4_O_9_ phase (67.8% phase for 100EDTA sample). All samples exhibited consistent band gap energies (2.25–2.28 eV), indicating visible light absorption. X-ray photoelectron spectroscopy (XPS) revealed increased oxygen vacancy concentrations in multiphase samples due to secondary phases and heterojunctions. Magnetic characterization showed an almost magnetic behavior in 33EDTA and 100EDTA (*M*_s_ of 0.0002 and 0.001 emu/g), with higher Bi_2_Fe_4_O_9_ content contributing to increased magnetism in 100EDTA. Photoluminescence (PL) measurements indicated higher electron–hole recombination in single-phase 33EDTA compared to multiphase samples with heterojunctions. Photocatalytic tests with methylene blue (MB) showed that at neutral pH, multiphase samples generally had higher, though still low, efficiency due to heterojunctions, degrading approximately 46% for the 100EDTA sample. Despite poor performance at neutral pH (32%), single-phase 33EDTA exhibited significantly improved photocatalytic activity at pH 3, achieving complete MB degradation after 120 min. This was attributed to the positive surface charge at lower pH, minimizing adsorptive effects. Mechanistic studies confirmed both electrons and holes are active in pure BiFeO_3_ photocatalysis, while heterojunctions primarily limited the mechanism to superoxide formation in multiphase samples.

## Graphical abstract

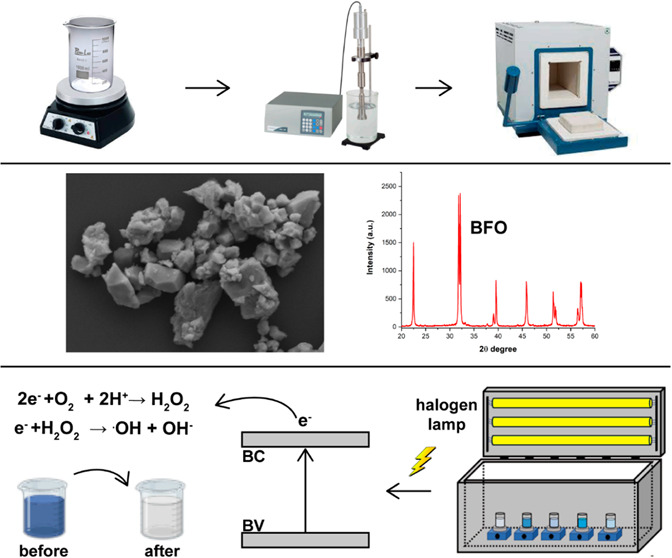

## 1 Introduction

The development of visible-light-responsive photocatalysts has garnered considerable attention due to the growing need for efficient solar energy utilization and environmental remediation technologies. Among various semiconductor materials, bismuth ferrite (BFO) has emerged as a promising candidate because of its unique combination of ferroelectric, magnetic, and photocatalytic properties.^1–3^ Bismuth ferrite can crystallize in multiple phases, including BiFeO_3_, Bi_25_FeO_40_, and Bi_2_Fe_4_O_9_, which adopt rhombohedral, cubic, and orthorhombic structures, respectively. This structural versatility enables the tuning of specific physical properties, particularly those associated with the rhombohedral BiFeO_3_ phase, which exhibits spontaneous electric polarization and antiferromagnetic ordering at room temperature-characteristics that qualify it as a multiferroic material.^4,5^

One of the most appealing features of BFO is its narrow bandgap (∼2.1–2.2 eV), which allows for strong absorption of visible light.^6,7^ In addition, the positions of the conduction and valence bands are suitably aligned with the redox potentials of water, making BFO a promising photoanode for water splitting in photoelectrochemical (PEC) cells.^8,9^ However, several limitations such as low electrical conductivity, high recombination rates of photogenerated carriers, and phase instability have hindered its practical photocatalytic applications.^10^

To address these challenges, numerous strategies have been proposed to enhance the optoelectronic performance of BFO. Structural modification through doping with transition metals or rare-earth elements has shown to improve charge carrier separation and suppress electron–hole recombination.^11–13^ Additionally, the formation of heterojunctions with other semiconductors-such as ZnO, graphene, and Bi_2_WO_6_ has been widely explored to create internal electric fields that facilitate spatial separation of charges and increase photocatalytic activity.^3,14,15^

Another critical factor influencing the photocatalytic performance of BFO is the presence of oxygen vacancies. These intrinsic point defects can act as donor states, improving n-type conductivity and facilitating charge transport. Experimental investigations coupled with density functional theory (DFT) simulations have demonstrated that oxygen vacancies in BFO lead to the formation of small polarons, which affect the material’s carrier mobility and recombination dynamics.^16,17^ Intentional introduction of oxygen vacancies via annealing under inert atmospheres has proven effective in increasing the majority carrier concentration and photocurrent generation in n-type BFO electrodes.^18^

Nanostructuring BFO into mesoporous or nanoscale morphologies has also emerged as a powerful strategy for performance enhancement. Mesoporous BFO/graphene composites, for instance, offer high surface areas and magnetic separability, allowing for facile catalyst recovery and improved reusability.^19,20^ These composites demonstrate significantly higher surface adsorption, light-harvesting efficiency, and degradation rates of contaminants such as methylene blue dye, compared to bulk BFO.^21^ Moreover, advanced synthetic techniques such as microwave-assisted hydrothermal processing have enabled the rapid and energy-efficient preparation of highly crystalline, uniformly distributed BFO nanoparticles, with reduced synthesis time and improved material quality.^22^ Other advanced techniques, such as the sonochemical method, have also proven to be effective for the synthesis of BFO nanoparticles.^23^

In summary, bismuth ferrite-based photocatalysts offer a promising platform for sustainable applications in environmental and energy-related fields. As discussed earlier, numerous studies have explored the optimization of BFO-based systems through combination with various materials. In this work, we investigate the use of EDTA as a chelating agent in the sonochemical synthesis of BFO systems with different phase compositions (BiFeO_3_, Bi_25_FeO_40_, and Bi_2_Fe_4_O_9_), and assess their resulting photocatalytic performance. Due to preliminary tests, EDTA was varied at 0, 33.33, 66.66, and 100%mol relative to iron nitrate.

## 2 Materials and Methods

### 2.1 Synthesis

To obtain bismuth ferrite powders, the following reagents were used: iron nitrate (Fe­(NO_3_)_3_·9H_2_O, Sigma-Aldrich, 98%), bismuth­(III) nitrate (Bi­(NO_3_)_3_·5H_2_O, Sigma-Aldrich, 98%), ammonium hydroxide (NH_4_OH–Synth, 30%), nitric acid (HNO_3_, Synth, 69%), and ethylenediaminetetraacetic acid (EDTA–C_10_H_14_N_2_O_2_Na_2_·2H_2_O, Dinamica).

Initially, a solution was prepared by dissolving 3.2 mmol of bismuth­(III) nitrate in 30 mL of deionized water, followed by the addition of 7 mL of nitric acid under constant stirring. Subsequently, a second solution was prepared by dissolving 3.2 mmol of iron nitrate in 30 mL of deionized water, also under continuous stirring. After complete dissolution of the reagents in both solutions, the second solution was poured into the first one and kept under agitation for 10 min. Then, 16 mL of ammonium hydroxide was added, adjusting the pH to 10, and the solution was stirred for another 10 min. The resulting solution was then subjected to ultrasonic treatment using an Ultronique Desruptor QR550 operating at a frequency of 20 kHz and a power of 550 W, equipped with a 13 mm ultrasonic tip, for 20 min. Finally, the supernatant was separated by centrifugation at 9000 rpm and then transferred to a calcination furnace, where it was heated at 750 °C for 4 h.

To obtain samples using EDTA as a chelating agent, the same procedure described above was repeated, with the only modification being the addition of EDTA in the second solution. The amount of EDTA was varied at 33.33%, 66.66%, and 100% of the mass of iron nitrate. Thus, the samples in this study were named according to the amount of EDTA used in their synthesis: 0EDTA, 33EDTA, 66EDTA, and 100EDTA.

### 2.2 Characterization

The obtained phases were characterized using X-ray diffraction (XRD) with a Shimadzu XRD-6000 instrument, using Cu Kα radiation (1.5418 Å). The scan angle ranged from 10° to 120°, with a scanning speed of 1°/min and a step size of 0.01°. To obtain more detailed crystallographic information on the synthesized samples, Rietveld refinement was performed using the General Structure Analysis System (GSAS) software with the EXPGUI interface.^24^

The samples were morphologically analyzed using a Hitachi TM-3000 field emission scanning electron microscope (FESEM) equipped with an energy-dispersive X-ray spectroscopy (EDX) detector for chemical characterization.

Diffuse reflectance spectroscopy in the ultraviolet–visible (UV–vis) region was performed using a Shimadzu UV-2600 spectrophotometer. The data were then converted into absorbance using the Kubelka–Munk function^25^ and the method proposed by Wood and Tauc^26^ was applied to estimate the band gap energy (*E*_gap_) of the samples.

The X-ray Photoelectron Spectroscopy (XPS) measurements were performed using a K-α spectrometer (Thermo Scientific) with an Al Kα X-ray source. The energy calibration was performed using the C 1s peak at 284.8 eV as a reference. Survey spectra were recorded to identify elemental composition, while deconvolution of core-level spectra was performed using a Voigt function with a Tougaard background subtraction.

Photoluminescence (PL) measurements were conducted using a Cobolt/Zouk laser operating at λ = 355 nm with a power of 20 mW. Signal detection was carried out with a silicon CCD detector (Andor–Kymera/Idus) coupled to a 19.3 cm spectrometer.

The point of zero charge (PZC) of the material was determined using the salt addition method with an inert electrolyte. For this, 50 mL solutions of NaCl at 0.01 mol·L^–1^ were prepared, and their initial pH values were adjusted to range from 2 to 13 using HCl and NaOH solutions, both at 0.1 mol·L^–1^. Then, 0.05 g of the solid material was added to each solution. The suspensions were kept under continuous stirring in closed containers for 24 h at room temperature (approximately 28 °C). After the contact time, the samples were filtered and the final pH values were measured. The PZC was estimated by plotting the pH variation (ΔpH = pH_final_ – pH_initial_) as a function of the initial pH. The pH value at which ΔpH equals zero was taken as the point of zero charge of the material.

The quasi-static magnetic characterization, at room temperature, was performed using a Lakeshore 7400 series Vibrating Sample Magnetometer (VSM) with a maximum magnetic field amplitude of 4 kOe. Due to the low magnetic moment of the studied samples, a rigorous calibration protocol was executed before the measurements. First, a previous calibration of the magnetic moment (emu) considering a pure Ni sample was executed. The empty sample holder was measured to verify any contamination. Finally, a known mass of powder sample was measured. For this procedure, masses of around 20 mg of each material were considered.

### 2.3 Photocatalytic Tests

The initial photocatalytic tests were conducted using the cationic methylene blue (MB) dye at neutral pH (pH 7). The procedure involved 50 mL of MB solution at a concentration of 10^–5^ mol/L and 0.05 g of the tested material. The dye-sample mixture was kept under constant stirring, and, in the absence of light, aliquots were collected every 10 min to monitor variations in dye absorbance. After three aliquots were withdrawn to analyze adsorptive effects, the MB solutions were then illuminated by a halogen lamp (118 mm, 500 W, with a UV filter). Subsequently, aliquots were taken at 20 min intervals. In total, six aliquots were collected under UV–C illumination. The withdrawn aliquots were analyzed using a Shimadzu UV-2600 spectrophotometer to assess changes in absorbance. To eliminate adsorptive effects during photocatalytic tests, adsorption tests were conducted for the same period of time. All adsorptive and photocatalytic tests were performed in triplicate, based on an average absorbance value for analyses.

To analyze the effect of reaction pH on the photocatalytic response, 0.1 M sodium hydroxide (99% purity, Neon) and 0.1 M hydrochloric acid (37% purity, ACS Cientifica) solutions were prepared. Then, 1 mL of these solutions was added to neutral pH MB dye solutions. This resulted in MB dye solutions at basic pH (pH 11) and acidic pH (pH 3). The point of zero charge (pH_pzc_) of the material was determined using the pH drift method. A series of 50 mL sodium chloride (99%, Exodo Cientifica) solutions (0.01 M) were prepared with initial pH values ranging from 2 to 12, adjusted using hydrochloric acid or sodium hydroxide (0.1 M). Then, 50 mg of the sample was added to each solution, and the suspensions were stirred for 24 h at room temperature. After equilibrium, the final pH values were recorded. The pH_pzc_ was defined as the point where the initial and final pH values were equal (ΔpH = 0), identified from the plot of ΔpH (pH_final_ – pH_initial_) versus pH_initial_.

Charge scavenger analysis was also performed to determine the mechanisms involved in the photocatalytic process of the synthesized samples. For this purpose, 1 mM silver nitrate (99.9% purity, Strem Chemicals) and 1 mM citric acid (99.5% purity, Synth) were added as scavengers for electrons (e^–^) and holes (h^+^), respectively, to the neutral pH MB dye solution.

The reusability tests were performed by physical separation of the photocatalyst powders via centrifugation. After separation, they were dried in an oven (100 °C) for 24 h and then subjected to new photocatalytic tests. This procedure was repeated three times, totaling four consecutive cycles.

## 3 Results and Discussion

The synthesized samples were characterized by XRD to identify the crystalline phases. The diffraction patterns obtained for the samples with varying amounts of EDTA are shown in Figure 1a. As observed in the diffractograms, the 33EDTA sample exhibits a visibly lower number of peaks, all corresponding to the BiFeO_3_ phase, with no formation of secondary phases. On the other hand, the remaining compositions show peaks associated with both BiFeO_3_ and the secondary phases Bi_25_FeO_40_ e Bi_2_Fe_4_O_9_. Obtaining pure BiFeO_3_ without secondary phases is challenging due to the metastability of this phase, which tends to form iron-rich (Bi_2_Fe_4_O_9_) or bismuth-rich (Bi_25_FeO_40_) phases.^27^ Kebede et al.^28^ synthesized BiFeO_3_ using a sol–gel method, followed by calcination at 550 °C for 2 h, and observed the appearance of Bi_2_Fe_4_O_9_ peaks as a secondary phase. Similarly, Han et al.^29^ synthesized BiFeO_3_ by a hydrothermal method, varying the synthesis temperature between 393 and 473 K, and detected secondary peaks corresponding to the Bi_25_FeO_40_ phase.

**Figure 1:**
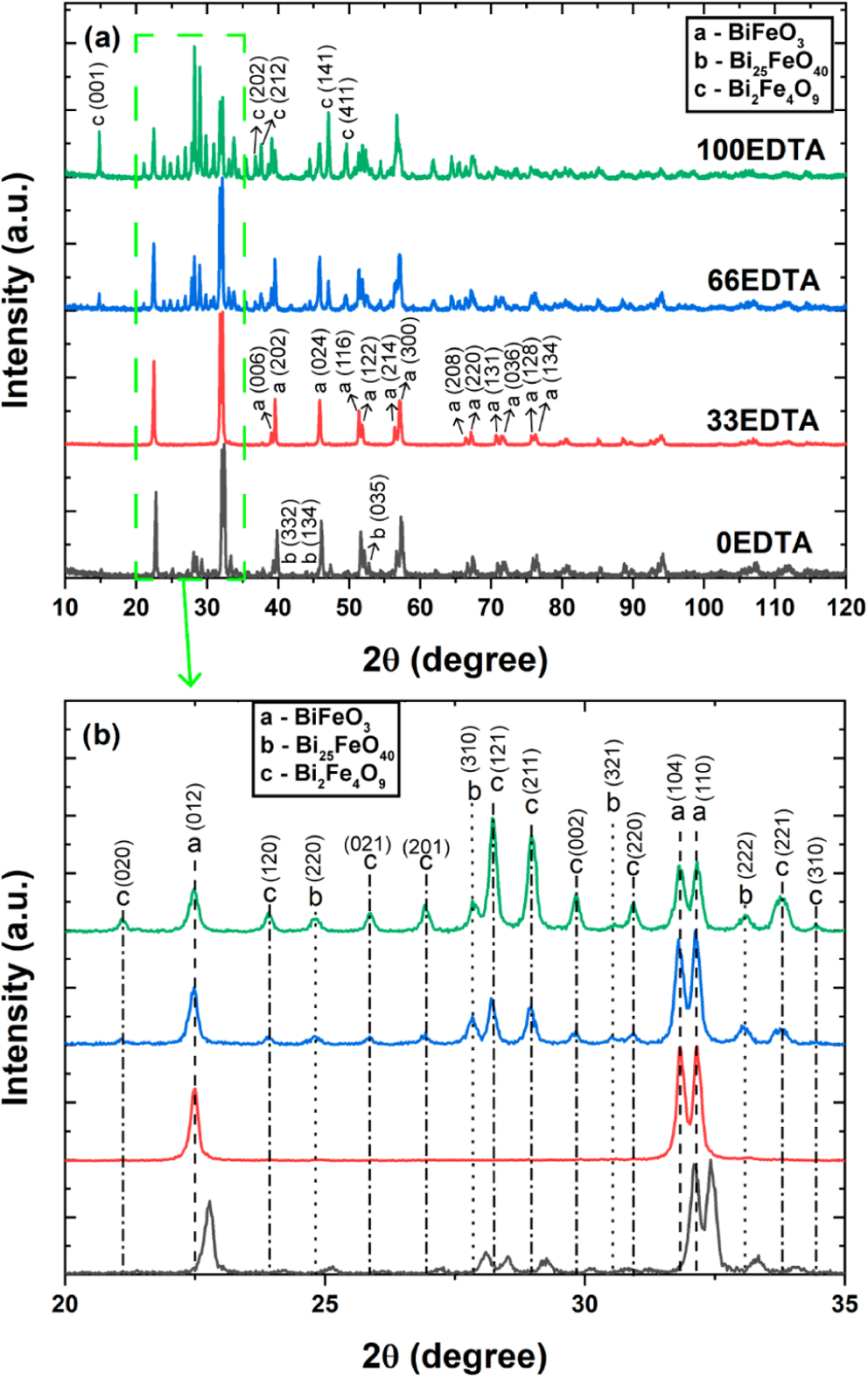
(a) Diffraction patterns of the synthesized samples and (b) magnified region highlighting phase identification.

Figure 1b presents a selected region of the diffraction pattern between 20° and 35°, highlighting the main peaks of the detected phases and indicating their respective attributions. The characteristic peaks of the BiFeO_3_ phase correspond to a rhombohedral structure with space group *R*3*c*, as referenced in ICSD card 1001090. The peaks associated with the Bi_25_FeO_40_ phase exhibit a cubic structure with space group *I*2_3_, as described in ICSD card 4030661, while the Bi_2_Fe_4_O_9_ phase presents an orthorhombic structure with space group *Pbam*, also referenced in ICSD card 1530918. To obtain more detailed crystallographic information on the samples, Rietveld refinement was performed. This refinement was based on adjustments of the scale factor, phase fraction, background (Chebyshev polynomial function), peak shape (Thomson-Cox-Hastings pseudo-Voigt), lattice parameters, atomic fractional coordinates, and isotropic thermal parameters. Figure 2 shows the comparative curves between experimental and theoretical diffraction patterns, while Table 1 presents the data obtained from the refinement.

**Figure 2:**
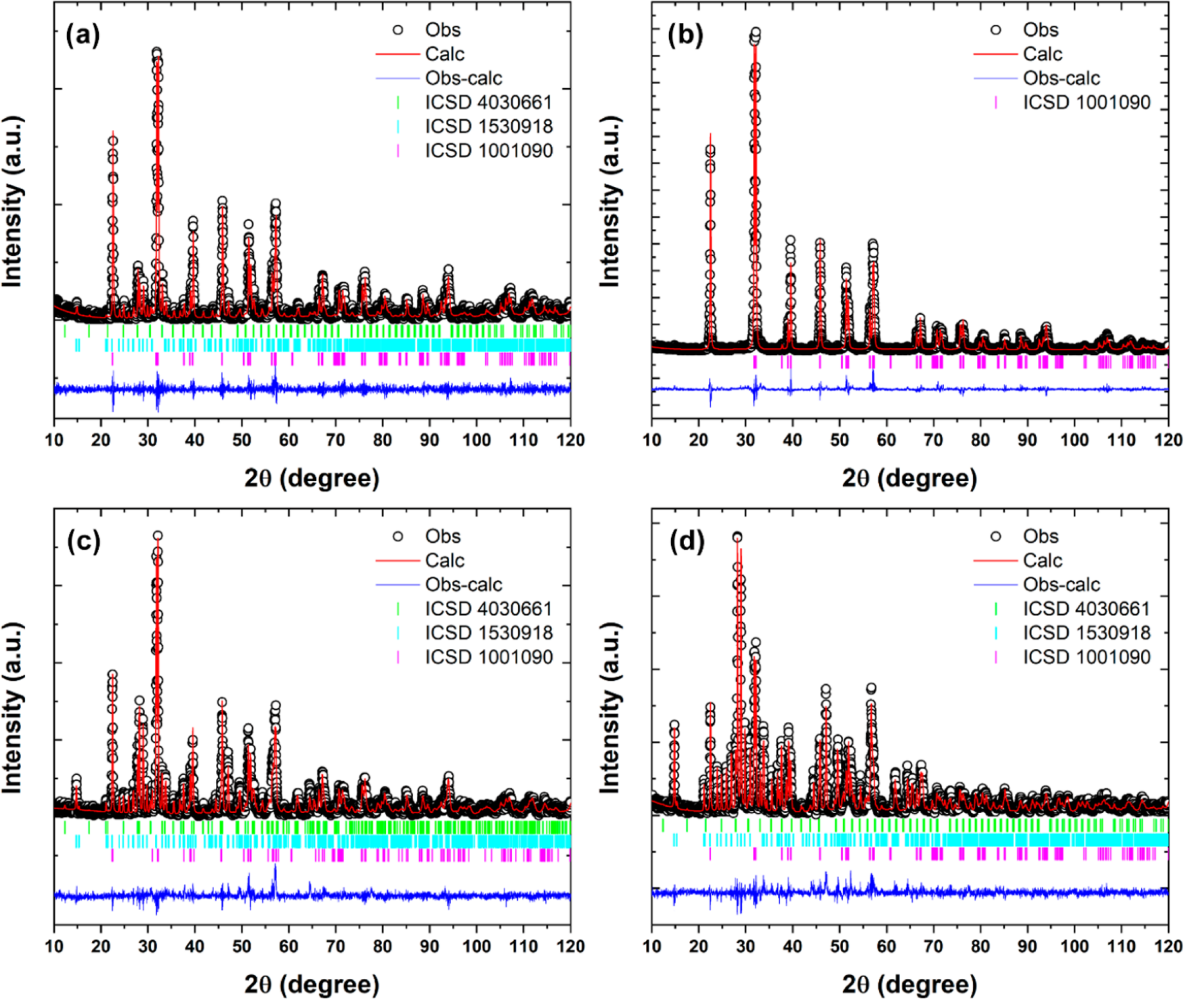
Calculated and observed diffraction patterns obtained from Rietveld refinement for (a) 0EDTA, (b) 33EDTA, (c) 66EDTA, and (d) 100EDTA samples.

**Table 1:** Microstructural and Quality Parameters Obtained by the Rietveld Refinement Using GSAS Software^a^

		0EDTA	33EDTA	66EDTA	100EDTA
BiFeO_3_	*a* (A)	5.5813	5.5748	5.5750	5.5759
	*c* (A)	13.8768	13.8604	13.8628	13.8675
	*V* (A^3^)	432.2749	430.7590	430.8645	431.1497
	%phase	68.94	100	55.19	25.42
	δ (nm)	48.1	58.1	53.2	47.4
	ε (10^–4^)	2.0	1.6	1.8	2.0
Bi_25_FeO_40_	*a* (A)	10.1674	-	10.1468	10.1457
	*V* (A^3^)	1051.0653	-	1044.6897	1044.3499
	%phase	10.66	-	9.30	6.75
	δ (nm)	41.8	-	38.3	40.0
	ε (10^–4^)	2.0	-	2.2	2.1
Bi_2_Fe_4_O_9_	*a* (A)	7.9672	-	7.9607	7.9582
	*b* (A)	8.4468	-	8.4389	8.4405
	*c* (A)	6.0010	-	6.0020	6.0013
	*V* (A^3^)	403.8514	-	403.2117	403.1144
	%phase	20.40	-	35.51	67.83
	δ (nm)	38.1	-	57.5	56.1
	ε (10^–4^)	2.2	-	1.5	1.5
refinement parameters	*W*_Rp_ (%)	24.96	18.81	22.87	19.14
	*R*_p_ (%)	17.98	13.38	16.85	14.45
	χ^2^ (%)	1.295	1.808	1.816	1.557
	*R*(*F* ^2^) (%)	12.79	8.18	9.34	9.65

a Where: δ = crystallite size and ε = microstrain.

As shown in Table 1, the refinement quality parameters (*W*_Rp_, *R*_p_, χ^2^, *R*(*F* ^2^)) exhibit satisfactory values, confirming the reliability of the obtained results. The table also indicates that for EDTA concentrations above 33.33%, the formation of the Bi_2_Fe_4_O_9_ phase is favored over the other phases. Along with the increased presence of this phase in the samples, there is also a corresponding increase in the average crystallite size.

EDTA was used in this research as a chelating agent, which has the function of forming stable complexes with metal ions to control their reactivity and homogeneity in solution. The carboxylate and amine functional groups present in the EDTA structure form stable intermediate complexes with the metal ions in the medium, reducing their reactivity and controlling rapid nucleation and particle growth.^30^ It is attributed that in the absence of EDTA, the precursor ions exhibit high reactivity, leading to heterogeneous and disordered nucleation. The low homogeneity of the medium favors the formation of secondary phases. For the 33EDTA quantity, it is considered the optimal amount, as its interaction with the metal ions reduces the medium’s reactivity, controlling the kinetics of formation and growth, and thus favoring the formation of BiFeO_3_ without the presence of secondary phases. Conversely, in the 66EDTA and 100EDTA samples, an excess of EDTA is attributed, where its action as a chelating agent becomes overly pronounced; it also interacts among themselves and not solely with the metal ions, creating steric hindrance and altering the formation kinetics of secondary phases. This effect can be further visualized by the increase in secondary phases with increasing EDTA concentration between the 66EDTA and 100EDTA samples, as shown by the data obtained from Rietveld refinement. A possible scheme for the formation of phases obtained by varying the amount of EDTA used is shown in Figure S1 (Supporting Information). The utilization of EDTA as a chelating agent in syntheses has been widely reported in the literature for obtaining various materials.^31–33^ Therefore, adjusting the EDTA concentration during the synthesis process is crucial for determining the resulting phase.

The morphology of the synthesized particles was analyzed using field emission scanning electron microscopy (FESEM), and the micrographs are shown in Figure 3. As observed in the FESEM images, all samples exhibit irregularly shaped particles with significant size variation. Additionally, it is evident that particle growth occurs in an oriented manner, where particles rearrange themselves so that similar planes align and grow to minimize surface energy, as indicated by the yellow arrows in certain regions. The growth mechanism proposed by Ostwald Ripening can be used to explain this phenomenon, where, through diffusion, smaller particles are consumed by larger ones to reduce the system’s overall energy.^34^ On the other hand, features resembling particle coalescence are also observed, characterized by the formation of necks between particles, particularly highlighted in Figure 3d,h.

**Figure 3:**
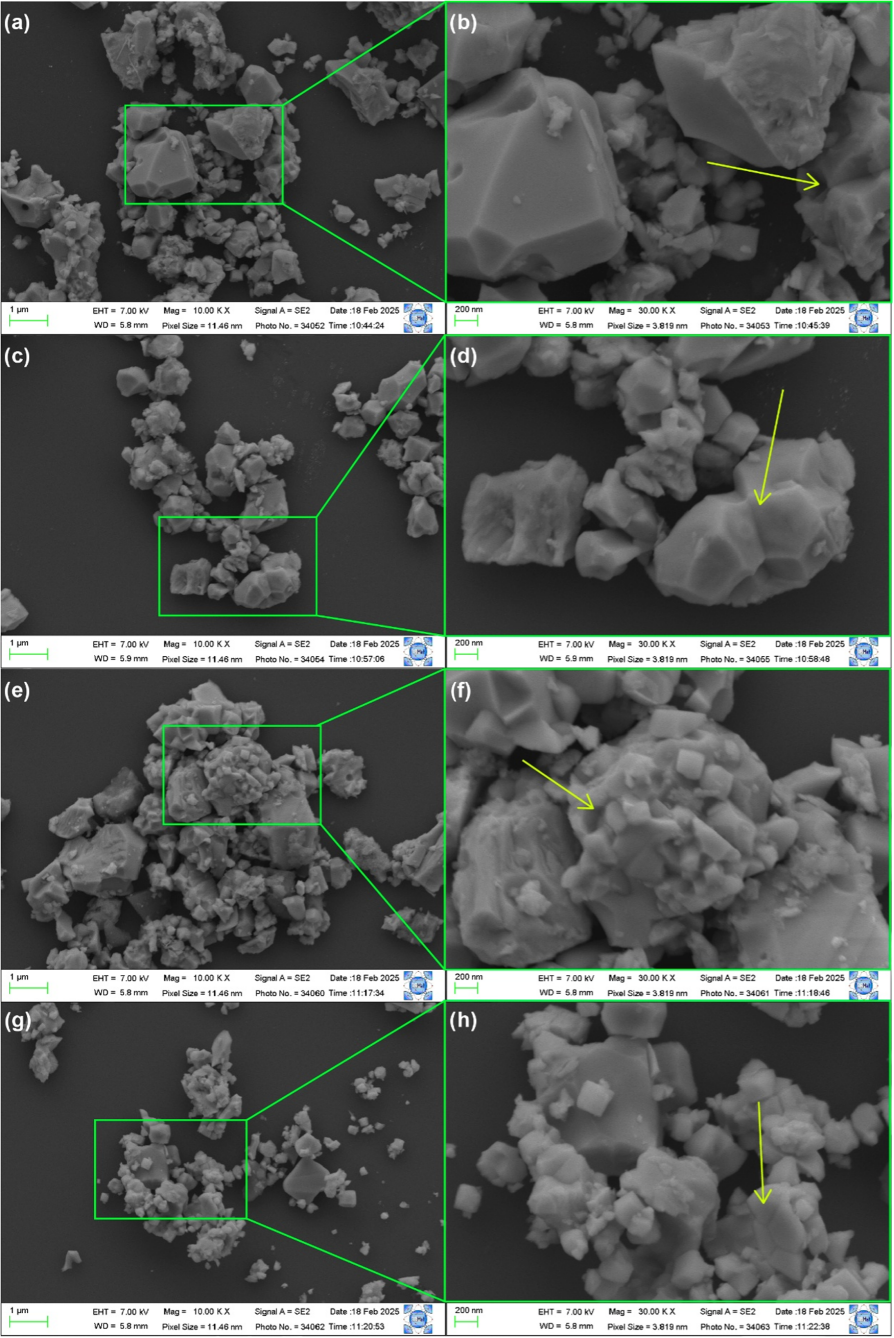
FESEM images for the (a, b) 0EDTA, (c, d) 33EDTA, (e, f) 66EDTA, and (g, h) 100EDTA samples.

Figure 4 presents the EDX spectrum and the chemical mapping performed on the 33EDTA sample. As shown by the results, the sample exhibits characteristic peaks corresponding to the Mα1 transition of bismuth, Lα1 of iron, and Kα1 of oxygen. The chemical mapping illustrates the uniform chemical distribution across all particles in the sample. This result is consistent with the XRD findings, where this sample only contains the BiFeO_3_ phase. The chemical similarity between the samples, along with the lack of well-defined morphology and the presence of multiple phases in the other samples, makes it difficult to differentiate the phases based on visible morphological aspects.

**Figure 4:**
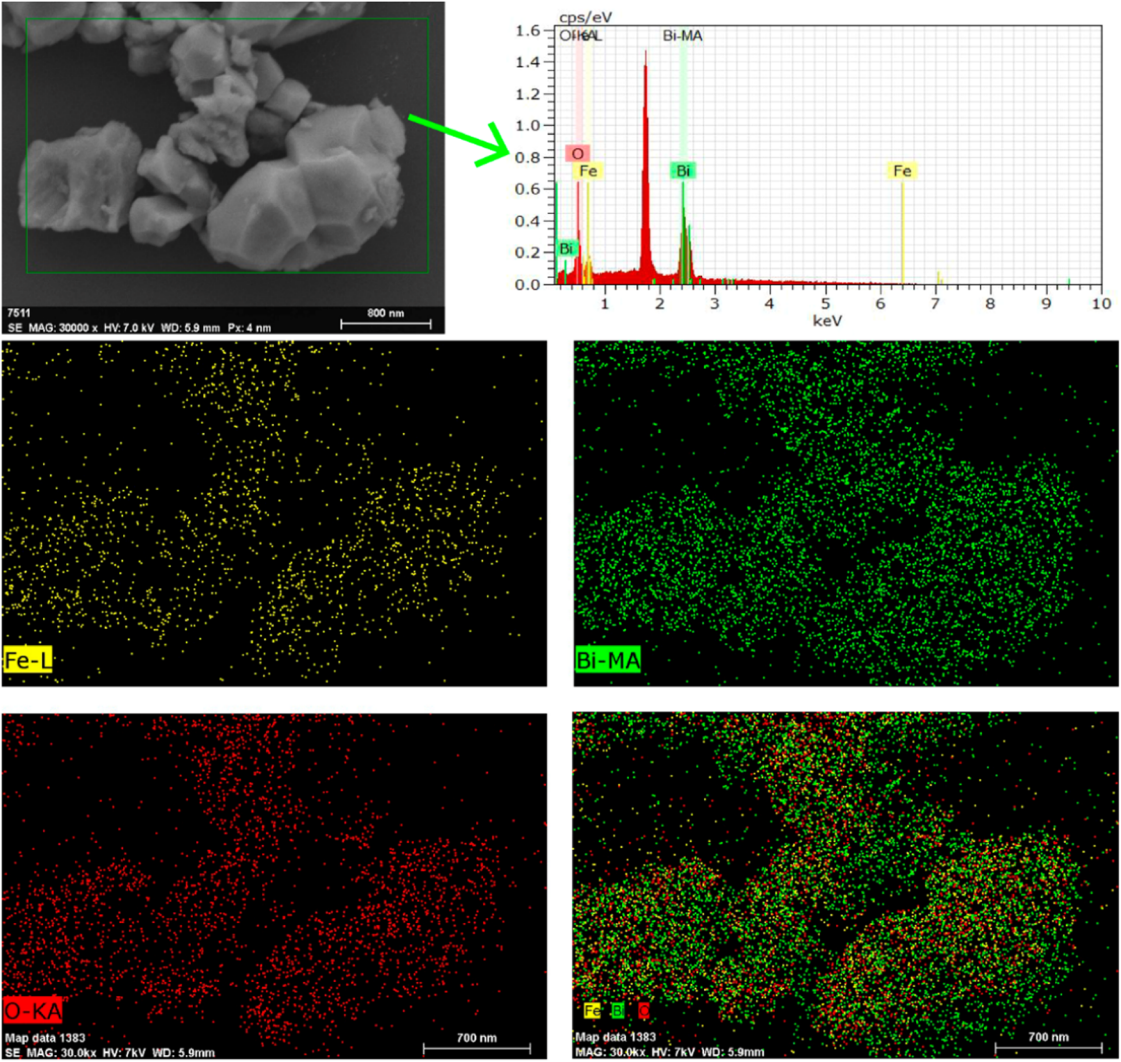
EDX chemical analysis and chemical mapping performed on the 33EDTA sample.

The synthesized samples were spectroscopically analyzed through diffuse reflectance, and the obtained data were then converted into absorbance using the methodology proposed by Kubelka–Munk. The curves are presented in Figure 5a. As seen in Figure 5a, the samples exhibit major absorbance peaks around 261, 347, and 441 nm. Additionally, they show less intense absorption bands in the region between 640 and 780 nm. These absorbance bands indicate that the material absorbs across the ultraviolet to infrared regions. The absorbance data were used to estimate the band gap (*E*_gap_) of the materials using the method proposed by Wood and Tauc for direct transitions. The resulting curves can be seen in Figure 5b–e for the 0EDTA, 33EDTA, 66EDTA, and 100EDTA samples, respectively. As shown in Figure 5b–e, despite the variation in the amounts of bismuth ferrite phases present in the samples, the Egap did not show significant changes, remaining between 2.25 and 2.28 eV. The values found in this study are consistent with those reported in the literature.^28,35^ These Egap values align with the absorbance range observed in Figure 5a, which is characteristic of a maximum absorption in the visible region.

**Figure 5:**
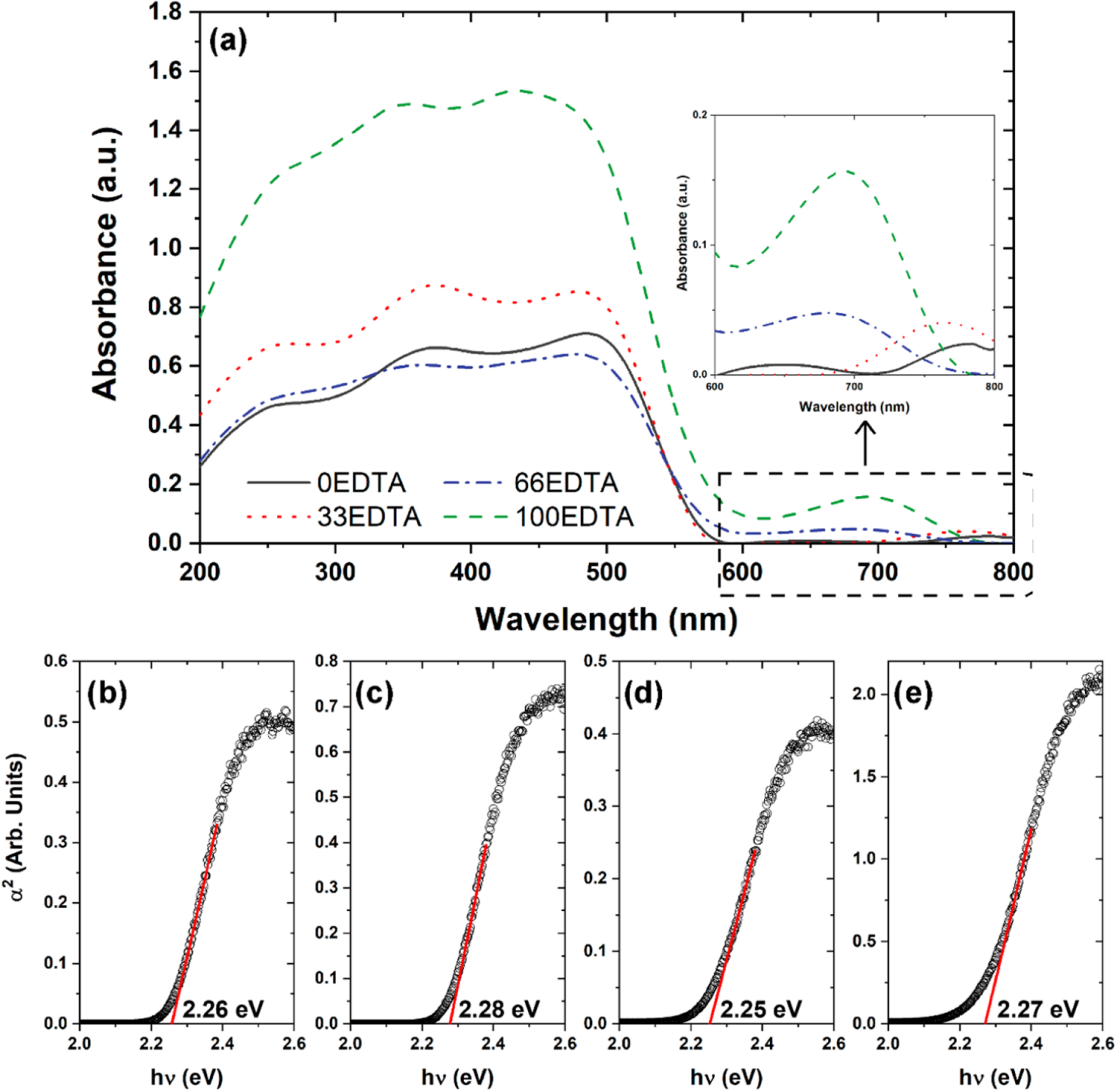
(a) Absorbance curves for all samples and (b–e) *E*_gap_ estimation using the direct transition for 0EDTA, 33EDTA, 66EDTA, and 100EDTA samples, respectively.

The XPS spectra of the 33EDTA and 100EDTA samples are shown in Figure 6. These samples were selected based on the phases present, those with the highest and lowest amounts of the BiFeO_3_ phase, respectively. This allows for the analysis of the chemical composition and elemental states of the compounds in the different phases. Figure 6a presents the full survey spectra for both samples, where peaks corresponding to Bi, Fe, and O in their various oxidation states can be observed. Figure 6b focuses on the Bi 4f region, showing peaks at 158.90 and 164.24 eV for the 33EDTA sample, which correspond to Bi 4f_7/2_ and Bi 4f_5/2_, respectively. In comparison, the 100EDTA sample exhibits these peaks at 158.62 and 163.97 eV, indicating a shift toward lower binding energies. The observed shift to lower binding energies may be attributed to an increased concentration of oxygen vacancies, which reduces the number of electronegative oxygen neighbors around Bi atoms, thereby decreasing the local electrostatic potential.^36^ Figure 6c displays the Fe 2p spectra. As shown, the Fe 2p peaks were deconvoluted into five fitted components. For the 33EDTA sample, the main peaks centered at 710.95 and 724.82 eV correspond to the Fe 2p_3/2_ and Fe 2p_1/2_ orbitals, respectively, and indicate the presence of Fe^3+^.^37^ In the 100EDTA sample, these peaks are shifted to slightly higher energies, appearing at 711.12 and 725.03 eV. Additional peaks obtained through fitting correspond to satellite structures, with the peak near 718 eV associated with the 2p_3/2_ orbital and those near 731, 734, 736, and 738 eV corresponding to the 2p_1/2_ orbital.^38^ Figure 6d shows the O 1s spectra. This peak can be deconvoluted into two components, which appear at 529.60 and 531.15 eV for the 33EDTA sample, and at 529.20 and 530.95 eV for the 100EDTA sample. The higher-energy peaks are typically attributed to adsorbed surface oxygen, while the lower-energy peaks are associated with lattice oxygen. The shift toward lower binding energies observed in the 100EDTA sample suggests an increase in oxygen vacancy concentration. This shift is expected due to the presence of secondary phases of bismuth ferrite, which promote the formation of heterojunctions and, consequently, a higher density of structural defects.

**Figure 6:**
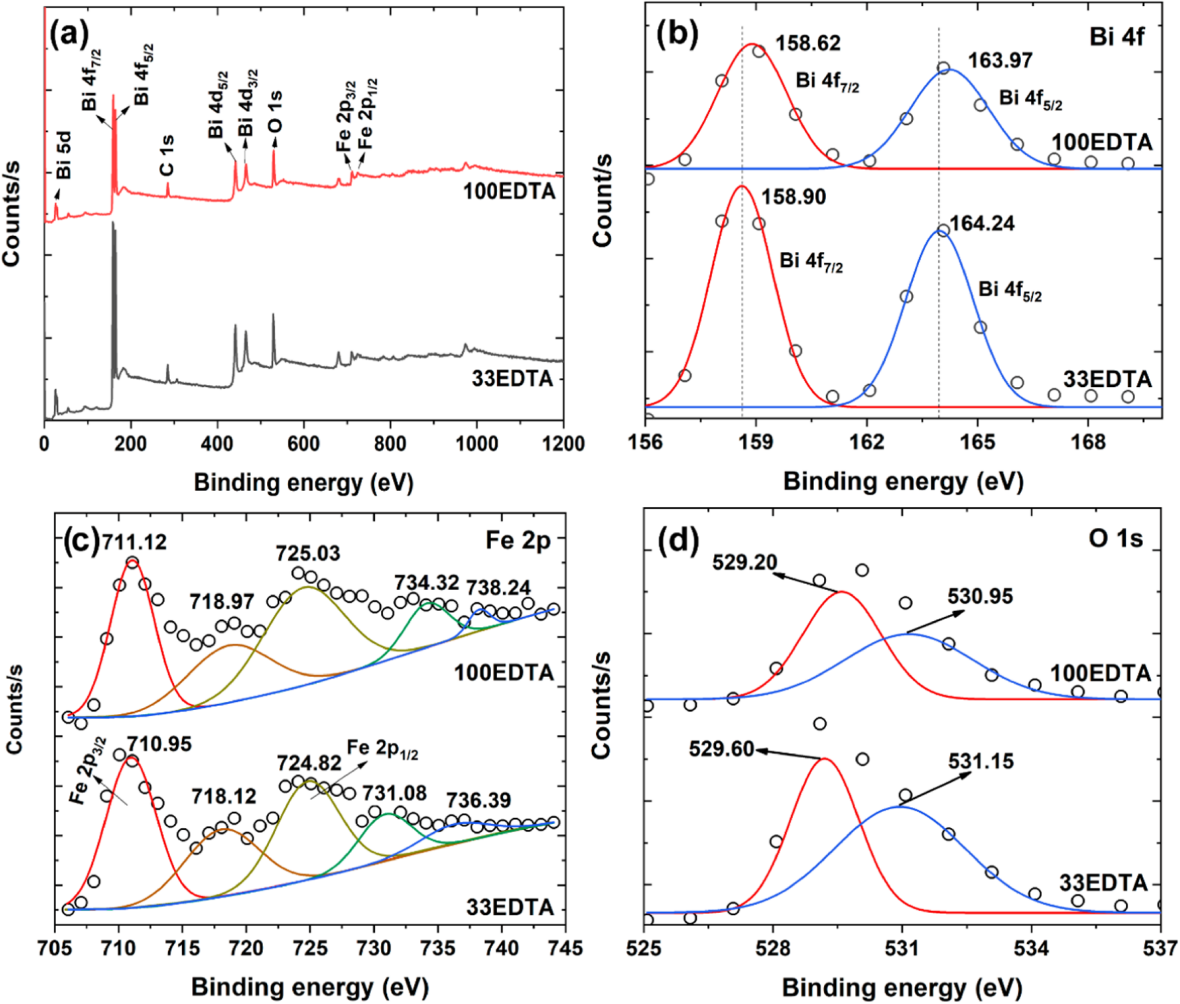
(a) Survey XPS spectra of the 33EDTA and 100EDTA samples, and high-resolution XPS spectra of (b) Bi 4f, (c) Fe 2p, and (d) O 1s regions.

The analysis of the magnetic properties of the samples was carried out through magnetization (*M*) versus magnetic field (*H*) curves at room temperature. Figure 7 presents the M-H curves for the synthesized samples, indicating a paramagnetic nature for the 0EDTA and 66EDTA samples. These results are consistent with those reported in the literature.^39,40^ Both 33EDTA and 100EDTA samples exhibit narrow S-shaped hysteresis loops, with saturation magnetizations (*M*_s_) of 0.0002 and 0.001 emu/g, and coercive forces of 0.04 and 0.06 kOe, respectively, indicating an almost magnetic behavior. The slight increase in the magnetic properties of the 100EDTA sample compared to the others may be associated with a higher amount of the Bi_2_Fe_4_O_9_ phase, which contains more Fe^3+^ ions in its crystal lattice. As previously discussed regarding the XPS analysis, the bands corresponding to Fe^3+^ can be observed, either through the main peaks or the satellite structures related to the Fe 2p_3/2_ and Fe 2p_1/2_ orbitals. The results obtained indicate that the synthesized samples in this study do not have sufficiently strong magnetic moments to induce an ordered behavior and retain their magnetic properties after the removal of the external field.

**Figure 7:**
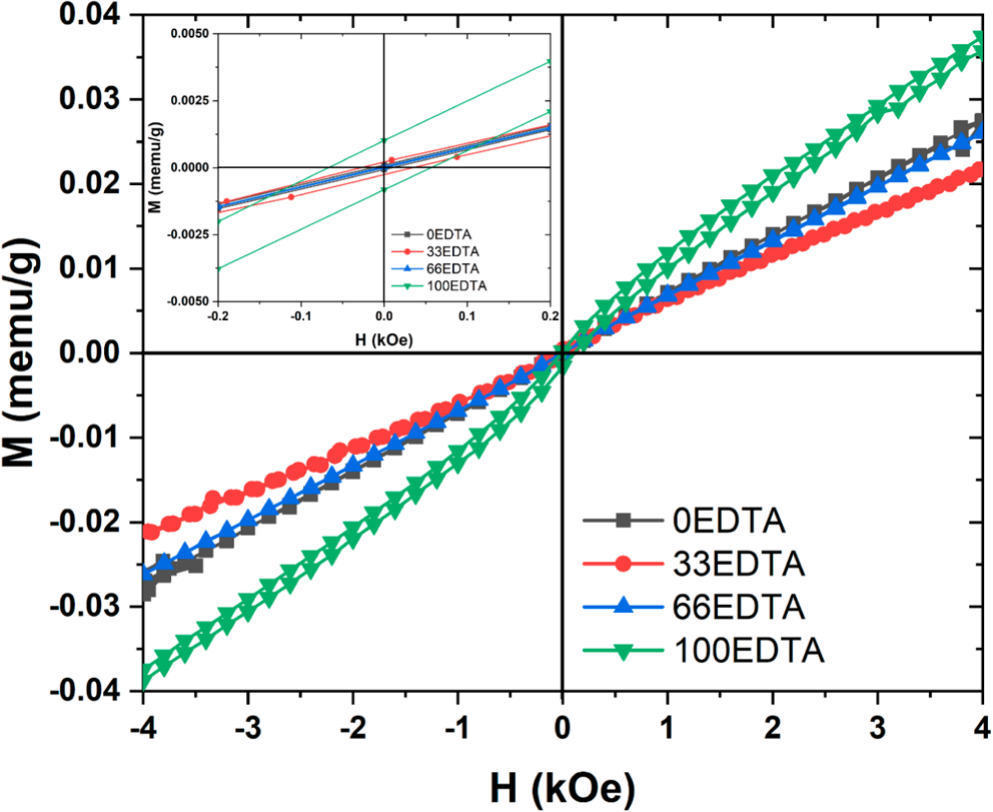
Magnetization (*M*) versus magnetic field (*H*) curves of the synthesized samples.

Photoluminescence (PL) measurements were conducted on the samples to obtain further optoelectronic information about the synthesized materials. Figure 8a presents the PL spectra acquired under excitation with a 355 nm laser. As observed, the 0EDTA sample exhibits a low photoluminescence emission intensity compared to the other samples. In contrast, the 33EDTA sample displays the highest relative intensity. A low photoluminescence intensity may be associated with a reduced recombination rate of electron–hole (e^–^/h^+^) pairs.^41^ As previously shown, the 33EDTA sample exhibits a single phase corresponding to BiFeO_3_. Consequently, no heterojunctions are present, unlike the other samples, which contain BiFeO_3_, Bi_25_FeO_40_, and Bi_2_Fe_4_O_9_ phases. The formation of heterostructures is widely reported in the literature as an effective strategy to suppress the recombination of photogenerated e^–^/h^+^ pairs.^42–44^ The 66EDTA and 100EDTA samples exhibit intermediate maximum intensities, with broad emission bands centered at 448, 553, and 768 nm for 66EDTA, and at 433, 601, and 805 nm for 100EDTA. The variations in the emission band positions can be attributed to the different proportions of phases present in the samples.

**Figure 8:**
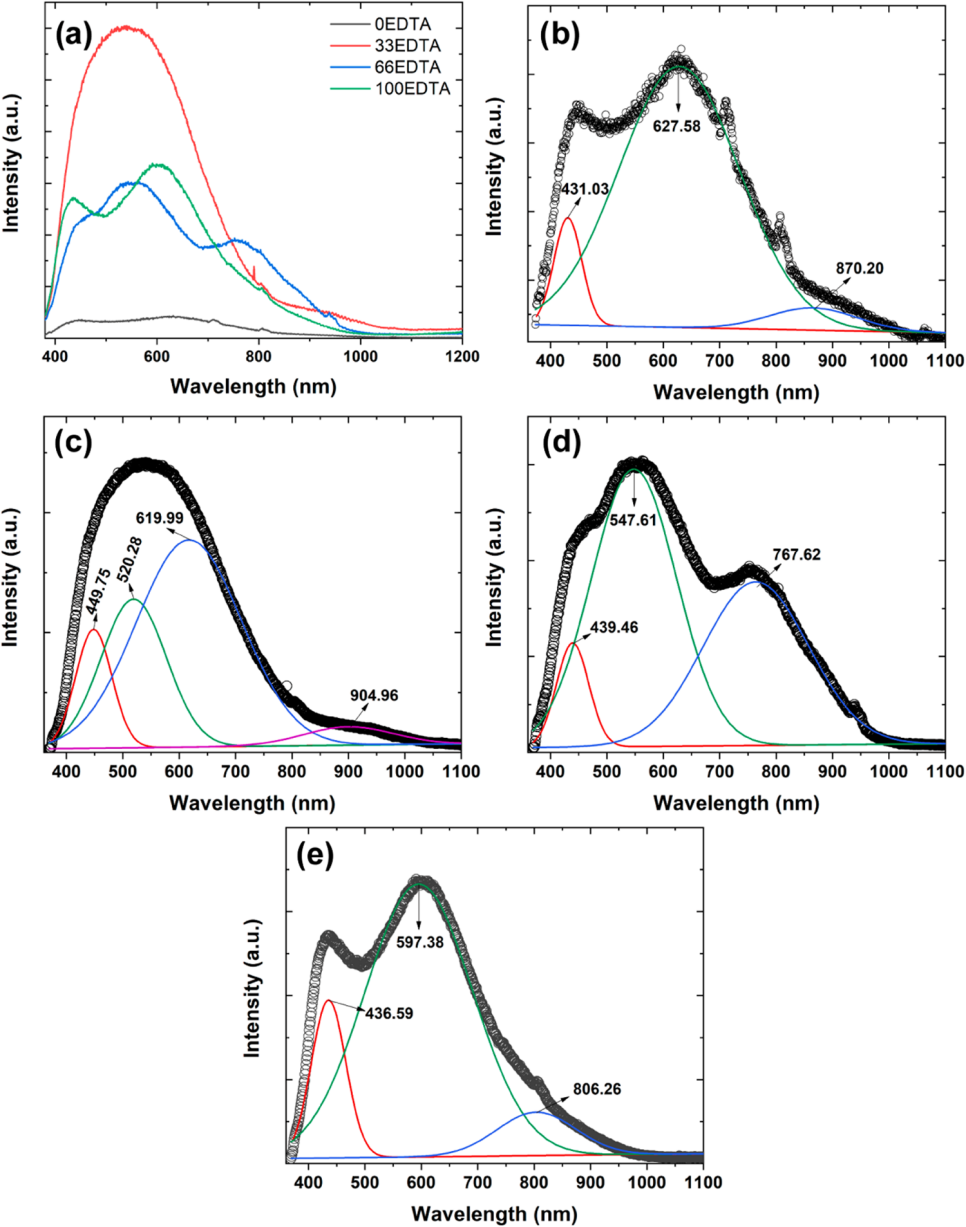
(a) Photoluminescence curves for the synthesized samples and PL curve deconvolution for the (b) 0EDTA, (c) 33EDTA, (d) 66EDTA, and (e) 100EDTA samples.

More detailed insights into the photoluminescent properties were obtained through the deconvolution of the PL curves, using Gaussian functions. The resulting curves are shown in Figure 8b–e. To achieve a better fit with the experimental spectra, the 0EDTA, 66EDTA, and 100EDTA samples were deconvoluted into three emission bands, while the 33EDTA sample required deconvolution into four bands. As previously discussed, all samples synthesized in this study exhibit a broad emission band covering the visible to infrared regions. As shown in Figure 8b, the 0EDTA sample displays a predominant emission centered around 627 nm. The 33EDTA sample (Figure 8c) shows a main emission near 620 nm, along with two additional significant bands around 449 and 520 nm, which also contribute notably to its PL spectrum. The 66EDTA sample (Figure 8d) exhibits two dominant emission bands, one centered around 547 nm and another near 767 nm. Finally, the 100EDTA sample (Figure 8e) shows a predominant emission around 597 nm.

The differences observed in the emission bands are closely related to the crystalline phases present in the samples. The 33EDTA sample, as previously shown, is composed exclusively of the BiFeO_3_ phase, and its main emission bands, ranging from the blue to orange regions, can be associated with shallow defects near the valence band.^45,46^ For the other samples, an increase in the Bi_2_Fe_4_O_9_ phase content correlates with enhanced emission intensities in the red and infrared regions. These longer-wavelength emissions are typically associated with deep-level defects.^47,48^ The absorbance spectra (Figure 5a) are consistent with these findings, as the 66EDTA and 100EDTA samples exhibit higher intensities near 700 nm, indicating the presence of intermediate energy levels further from the valence band compared to the other samples.

The photocatalytic properties of the samples were tested against methylene blue (MB) dye under simulated visible radiation using a halogen lamp with a UV filter. The obtained absorbance data were processed such that each withdrawn aliquot was denoted as *C*, and the initial dye absorbance, without any contact with the materials and/or radiation, was denoted as *C*_0_. Then, *C*/*C*_0_ curves were plotted, as shown in Figure 9. Adsorption tests, conducted on the 33EDTA and 100EDTA samples (which exhibited the best and worst photocatalytic results, respectively), are presented in Figure S2 (Supporting Information). The curves demonstrate that these materials achieve saturation, maintaining a constant dye concentration. This ensures that the photocatalytic effects can be accurately measured in isolation. Figure 9a presents the *C*/*C*_0_ curves for the samples tested against the MB dye at neutral pH (pH 7). As can be seen, generally, the samples exhibit low efficiency, with the 100EDTA sample showing the best result, reducing approximately 46% of the MB dye concentration. Furthermore, the 33EDTA sample showed the worst result, reducing approximately 32% of the MB dye concentration. It is also worth noting that even though the 100EDTA sample presented the best photocatalytic response, there are no significant differences compared to the 0EDTA and 66EDTA samples. As previously discussed, the 33EDTA sample contains only the BiFeO_3_ phase, while the other samples also present the Bi_25_FeO_40_ and Bi_2_Fe_4_O_9_ phases. Thus, the improved photocatalytic response can be associated with the heterojunctions formed between the phases, which reduce the recombination of electron/hole (e^–^/h^+^) pairs, corroborating the photoluminescence spectra. The increase in photocatalytic efficiency with the formation of heterostructures is widely reported in the literature.^49–51^

**Figure 9:**
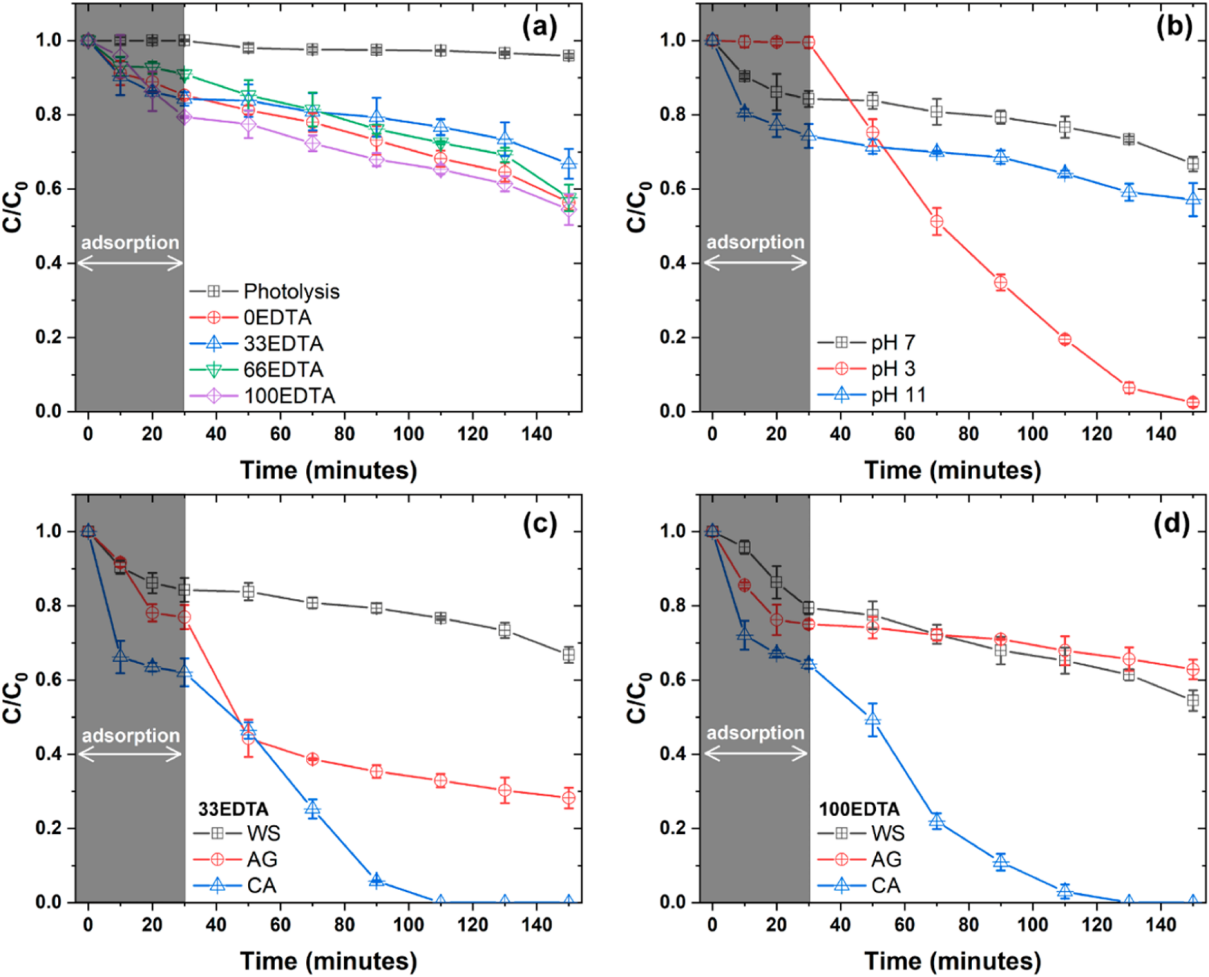
*C*/*C*_0_ curves for (a) MB tests at neutral pH and (b) varying pH for the 33EDTA sample. (c, d) Show the scavenger methodology results for 33EDTA and 100EDTA samples, where WS = without scavengers, AG = AgNO_3_, and CA = citric acid.

As observed, sample 33EDTA exhibits the lowest photocatalytic activity against MB dye at neutral pH. However, this sample is the only one that presents a single phase. Therefore, it was tested at different reaction pH values, as shown in Figure 9b. As can be seen, reducing the reaction pH to 3 inhibited the adsorptive effects, while at pH 11, an increase was observed. On the other hand, both pH variations, whether to the acidic or basic medium, improved the photocatalytic activity of sample 33EDTA. The increase was more significant at pH 3, where the sample completely degraded methylene blue after 120 min of testing. A better understanding of the pH alterations in the adsorptive/photocatalytic properties of sample 33EDTA can be obtained by analyzing the point of zero charge (pH_pzc_). The curve obtained from the pH_pzc_ analysis can be seen in Figure 10. Based on the results, the sample has a pH_pzc_ = 6.51. Thus, for pH values lower than the pH_pzc_, the sample is positively charged, while for media with a pH higher than the pH_pzc_, the sample becomes negatively charged.^52,53^ Therefore, it can be attributed that at pH 3, due to the positive surface charge of the particles, there are no significant interactions for adsorption to occur.

**Figure 10:**
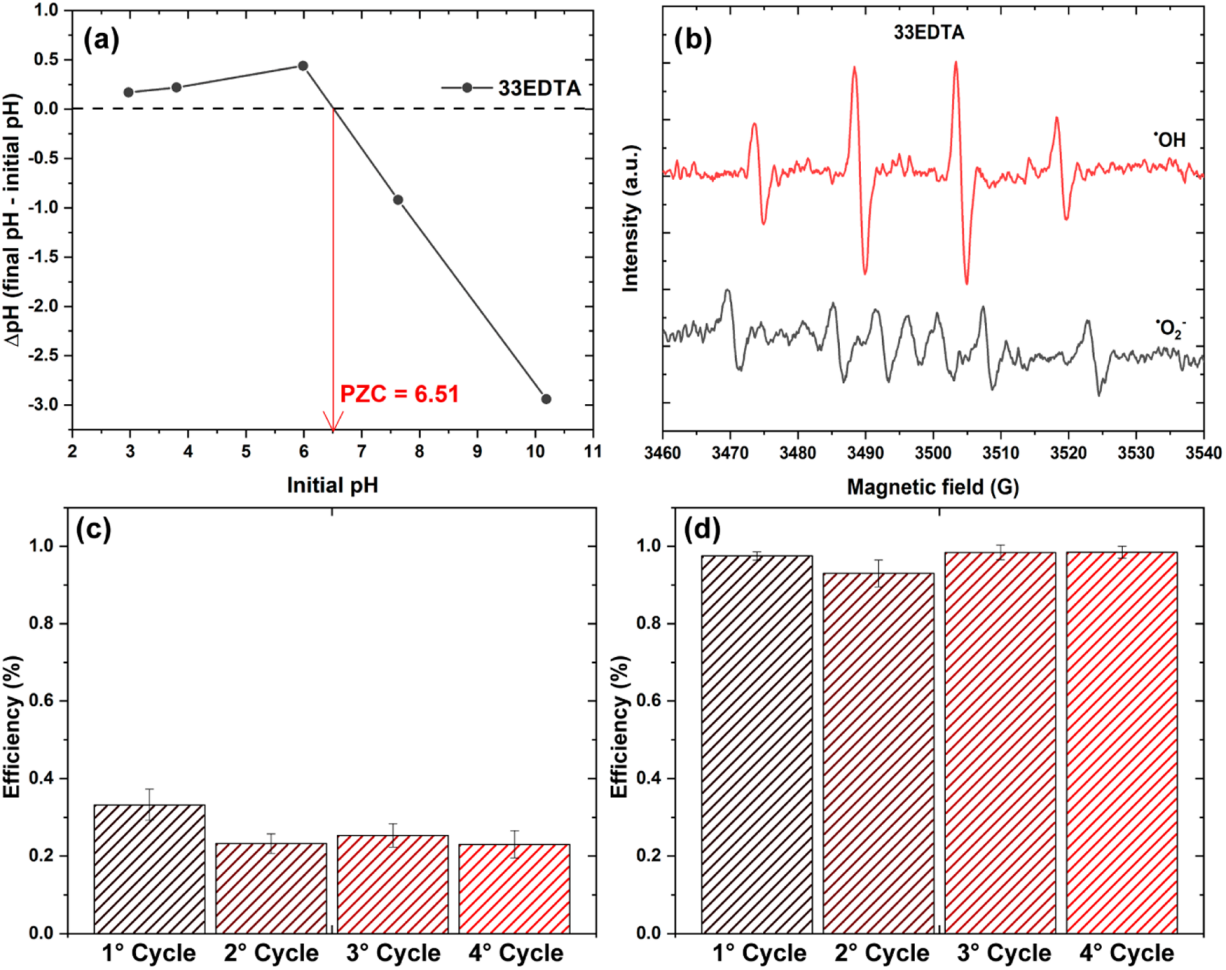
(a) ΔpH versus initial pH curve for pH_pzc_ determination, (b) ESR experiments for ^•^OH and O_2_^•–^ capture experiments, and reuse tests for 33EDTA sample at (c) pH 7 and (d) pH 3.

Another crucial factor for understanding the photocatalytic results lies in comprehending the active mechanisms. Thus, silver nitrate and citric acid were used as scavengers for electrons (e^–^) and holes (h^+^) at neutral pH in samples 33EDTA and 100EDTA, which are the samples exclusively containing BiFeO_3_ and the one with its lowest quantity, respectively. For 33EDTA, the addition of both scavengers positively influences the photocatalytic efficiency. In contrast, for 100EDTA, only citric acid has a positive effect. These results indicate that for 33EDTA, both charge carriers play a significant role in the photocatalytic process, with electrons predominantly acting in reduction reactions and the consequent formation of superoxide (O_2_^•–^) do not play a significant role. Knuth et al.^54^ showed that for BiFeO_3_, both electrons and holes play a crucial role in photocatalysis against rhodamine-B. These findings indicate that while the BiFeO_3_ phase involves both superoxide formation and hydroxyl radicals as active mechanisms in photocatalysis, the presence of other phases and, consequently, heterojunctions, limits the active mechanism primarily to superoxides.

To gain a deeper understanding of the BFO samples’ performance in the photocatalytic process, the energy calculations for the valence band (VB) and conduction band (CB) were performed using eqs 1 and 2, as proposed by Mulliken’s^55^

1 $$E_{\mathrm{CB}}=X-E_{0}-0.5E_{g}$$

2 $$E_{\mathrm{VB}}=E_{\mathrm{CB}}+E_{g}$$

Where: *E*_g_ is the bandgap energy of the semiconductor, *E*_0_ is the energy of free electrons on the hydrogen scale (4.5 eV) and χ is the absolute electronegativity of the semiconductor. To determine the absolute electronegativity, the electronegativities of the elements (χ_Bi_ = 4.69, χ_Fe_ = 4.06, and χ_O_ = 7.54) and their molar quantities were considered.^56^ Thus, the electronegativities of the three phases were determined as 6.06 eV/NHE for BiFeO_3_, 6.00 eV/NHE for Bi_2_Fe_4_O_9_, and 6.24 eV/NHE for Bi_25_FeO_4_. For a better approximation, the absolute electronegativity of the samples was estimated using the phase quantities present in the samples, as obtained from Rietveld refinement results, so that 0EDTA, 33EDTA, 66EDTA, and 100EDTA samples have χ values of 6.07, 6.06, 6.05, and 6.03 eV/NHE.

The VB and CB energies for the samples are shown in Table 2. As per the presented data, there are no significant changes even with the variation of the present phases. Such data occur due to the proximity of the Egap and the electronegativity of the samples. The CB energies are close to 0.42 V, while the VB energies are close to 2.70 V. These results are consistent with those found in the literature.^57^ The potentials of the valence and conduction bands are of paramount importance for the formation of superoxide radicals (O_2_^•–^) and hydroxyl radicals (^•^OH). The main equations governing photocatalytic processes are shown in eqs 3 to 9.

**Table 2:** Energies of the Valence and Conduction Bands for the Samples Synthesized in This Work

sample	conduction band (CB)	valence band (VB)
0EDTA	0.44 V	2.70 V
33EDTA	0.42 V	2.70 V
66EDTA	0.43 V	2.68 V
100EDTA	0.40 V	2.67 V

3 $$\mathrm{BFO}+\mathrm{hv}\to \mathrm{BFO}\left(e^{-}+h^{+}\right)$$

4 $$h^{+}+H_{2}O\to {\mathrm{OH}}^{•}+H^{+}$$

5 $$h^{+}+OH^{-}\to OH^{•}$$

6 $$e^{-}+O_{2}\to O_{2}^{•-}$$

7 $$2e^{-}+O_{2}+2H^{+}\to H_{2}O_{2}$$

8 $$H_{2}O_{2}+\mathrm{hv}\to 2{\mathrm{OH}}^{•}$$

9 $$e^{-}+H_{2}O_{2}\to OH^{•}+{\mathrm{OH}}^{-}$$

Equation 3 presents a theoretical form for the process of e^–^/h^+^ pair generation through the absorption of sufficient energy. The photogenerated pairs then react with species in the medium to form reactive oxygen species (ROS), which will degrade the MB dye. Equations 4 and 5 show the main ways h^+^ act in the generation of hydroxyl radicals, which are the species with the highest oxidative capacity.^58^ As shown previously, the synthesized samples exhibit a potential sufficiently higher than required for ^•^OH formation (∼2.38 V), thus being thermodynamically viable. On the other hand, eqs 6 to 9 show the action of e^–^ in the generation of ROS. In turn, the conduction band potential of the synthesized samples is not sufficient for the direct formation of O_2_^•–^ (∼ −0.33 V), making eq 6 thermodynamically unviable. However, considering that electrons are the main actors in photocatalytic processes, it can be attributed that the primary mechanisms involved in the photocatalysis of MB dye for the synthesized samples are characterized by eqs 7 to 9.

The formation of H_2_O_2_ through the action of e^–^ is thermodynamically favorable at CB potentials lower than 0.695 V. Equation 8 presents one of the methodologies in which ^•^OH formation occurs from the effect of radiation energy on H_2_O_2_. Equation 9 presents a secondary form of H_2_O_2_ breakdown; however, for its occurrence to be thermodynamically favorable, the CB needs to have a potential lower than 0.38 V. Ahmad et al.^59^ tested the photocatalytic properties of BiFeO_3_ nanoparticles against Congo Red dye, reducing its concentration by approximately 28% after 24 min of testing. This result was associated with the generation of ^•^OH radicals, which is in agreement with the mechanism proposed in this work. Tong et al.^60^ synthesized BiFeO_3_ disks by a hydrothermal method and tested them against methyl orange (MO) dye and phenol. Their results indicate that the photocatalytic activity of BFO is directly related to the presence of H_2_O_2_ in the reaction medium, which corroborates the mechanism proposed in this work for the degradation of MB dye. Shakir et al.^35^ performed photocatalytic tests of pure BiFeO_3_, Gd^3+^ doped, and g-C_3_N_4_ decorated BiFeO_3_ against MB dye and the anti-inflammatory Ibuprofen. The results obtained by them indicate that pure BiFeO_3_ has a photocatalytic efficiency of 53% after 120 min, while both doping and decoration act to increase its efficiency due to improved charge separation. They further concluded that e^–^ play the main role in the photocatalytic activity of the synthesized samples.

The proposed photocatalytic mechanisms are in agreement with the ESR results obtained for the 33EDTA sample. As can be seen in Figure 10b, there is generation of DMPO–OH^•^ and DMPO-O_2_^•–^ radicals, with the latter being of lower intensity. Although standard equations indicate that the generation of O_2_^•–^ is thermodynamically unviable for the proposed absolute model, the presence of intermediate levels originating from defects acts to increase the negativity of the conduction band potential, generating these species via an alternative pathway.

In photocatalytic processes, it is of utmost importance to understand the behavior of the photocatalyst not just in a single cycle, primarily due to the complexity and costs associated with the process. Thus, it becomes essential to analyze the reusability capacity of these semiconductors. In this work, reusability tests were performed for 4 consecutive cycles, without any thermal and/or chemical treatment between them, and the efficiency results are shown in Figure 10c,d. As can be seen, there are no significant variations in the photocatalytic efficiency of the samples at both pH 7 and pH 3, the latter having shown the highest efficiency. Knuth et al.^54^ analyzed the photocatalytic activity of BiFeO_3_, obtained by a hydrothermal method, against rhodamine-B dye and showed that the samples maintain their photocatalytic efficiency even after 3 cycles. On the other hand, Wu et al.^61^ showed that BiFeO_3_ synthesized by a hydrothermal method lost approximately 20% of its photocatalytic efficiency against methylene blue dye between the first and fourth cycles, even without apparent structural changes. The results obtained in this work serve as insights for future studies aiming to synthesize BiFeO_3_ without secondary phases, as well as for delving deeper into its photocatalytic properties.

## 4 Conclusions

This study successfully synthesized bismuth ferrite systems with varying phase compositions using an EDTA-assisted sonochemical method, demonstrating the critical role of EDTA concentration in controlling phase purity. The single-phase BiFeO_3_ exhibited distinct photocatalytic behavior compared to the multiphase samples. While heterojunctions in multiphase samples (like 100EDTA) generally enhanced photocatalytic efficiency by reducing electron–hole recombination at neutral pH, the single-phase 33EDTA sample showed superior photocatalytic performance under acidic conditions (pH 3). This enhancement was attributed to the positive surface charge of the particles at lower pH values, which minimized undesirable adsorptive effects. Furthermore, mechanistic studies revealed that both electrons and holes significantly contribute to the photocatalysis in the pure BiFeO_3_ phase, primarily through the formation of superoxide radicals. In contrast, the presence of other phases and heterojunctions in the multiphase samples appears to limit the active photocatalytic mechanism predominantly to superoxides. These findings provide valuable insights into controlling the phase composition of bismuth ferrites and tuning their photocatalytic mechanisms through pH adjustments and the presence of heterojunctions, which can guide the design of more efficient photocatalytic materials for environmental remediation.

## Supplementary Material



## Notes

Scheme of EDTA action as a chelating agent in the synthesis of the (a) 0EDTA, (b) 33EDTA, and (c) 66EDTA and 100EDTA samples (Figure S1), and adsorption tests performed for 150 min using methylene blue (MB) dye for the 33EDTA and 100EDTA samples (Figure S2) (PDF)
