# Supplementary material for: EDTA-Assisted Sonochemical Synthesis of Polymorphic Bismuth Ferrites: Structural and Photocatalytic Characterization
Source: ACS Omega. 2025 Aug 27;10(35):40364–78. doi: 10.1021/acsomega.5c05699 (PMC12423843; doi:10.1021/acsomega.5c05699)
Supplement: Supplementary file 1 [file ao5c05699_si_001.pdf]

EDTA-assisted sonochemical synthesis of polymorphic bismuth ferrites: structural and photocatalytic characterization

Nivaldo Freire de Andrade Neto[1*], Joyce Marina Paiva da Silva[1], João Marcelo Soares da Cunha[1], Marcio Daldin Teodoro[3], Marcio Assolin Corrêa[2], Mauricio R.D. Bomio[1], Fabiana Villela da Motta[1].

[1]LSQM – Laboratory of Chemical Synthesis of Materials – Department of Materials Engineering, Federal University of Rio Grande do Norte – UFRN, P.O. Box 1524, Natal-RN, Brazil.
[2]Physics Department, Federal University of Rio Grande do Norte, 59078-900 Natal, RN, Brazil
[3]Department of Physics, Federal University of São Carlos, 13565-905, São Carlos, SP, Brazil.

***Corresponding author:**
E-mail address: nivaldo.neto@ufrn.br (N. F. Andrade Neto)

1. Supplementary materials

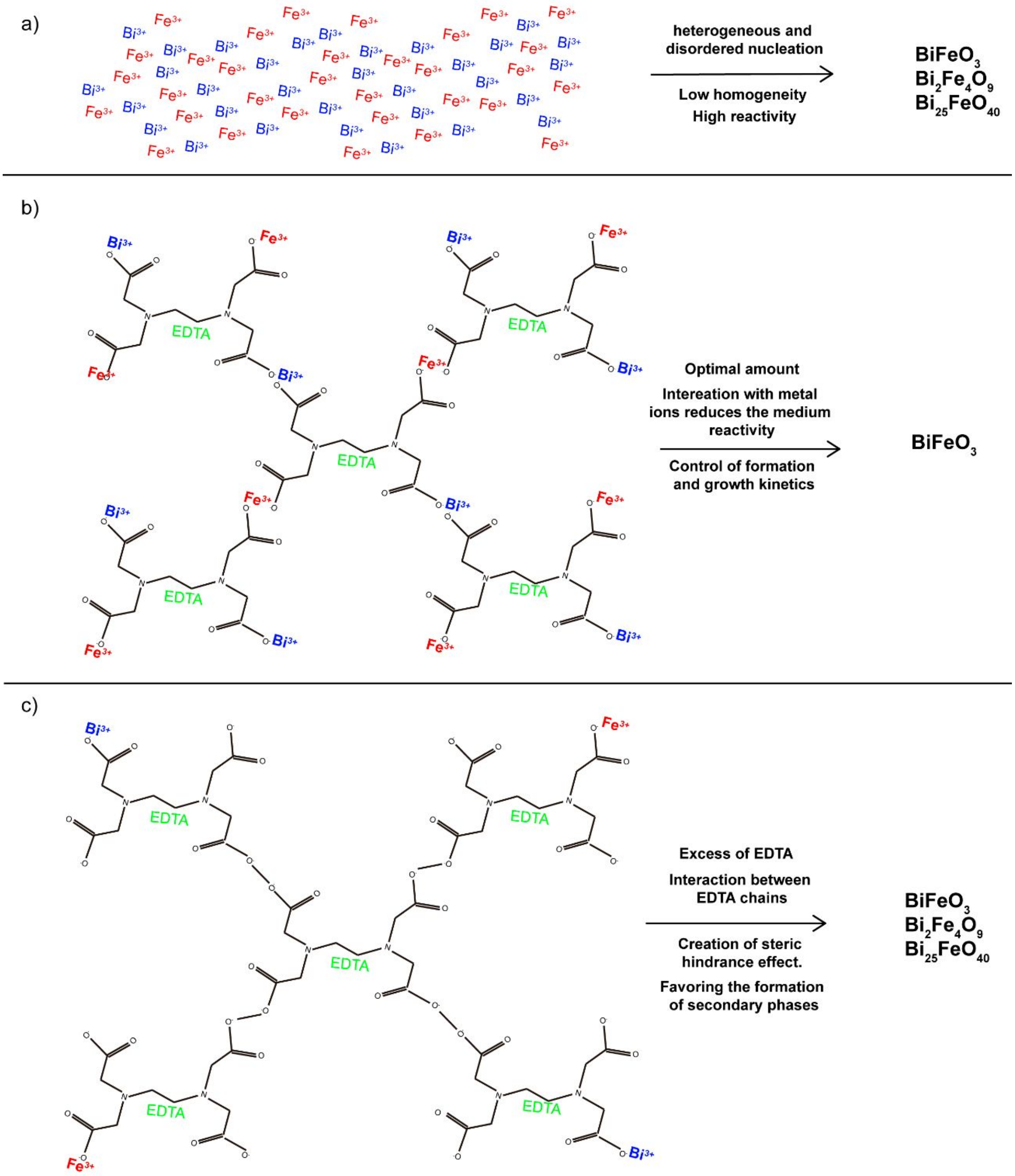

Figure S1. Scheme of EDTA action as a chelating agent in the synthesis of the (a) 0EDTA, (b) 33EDTA, and (c) 66EDTA and 100EDTA samples.

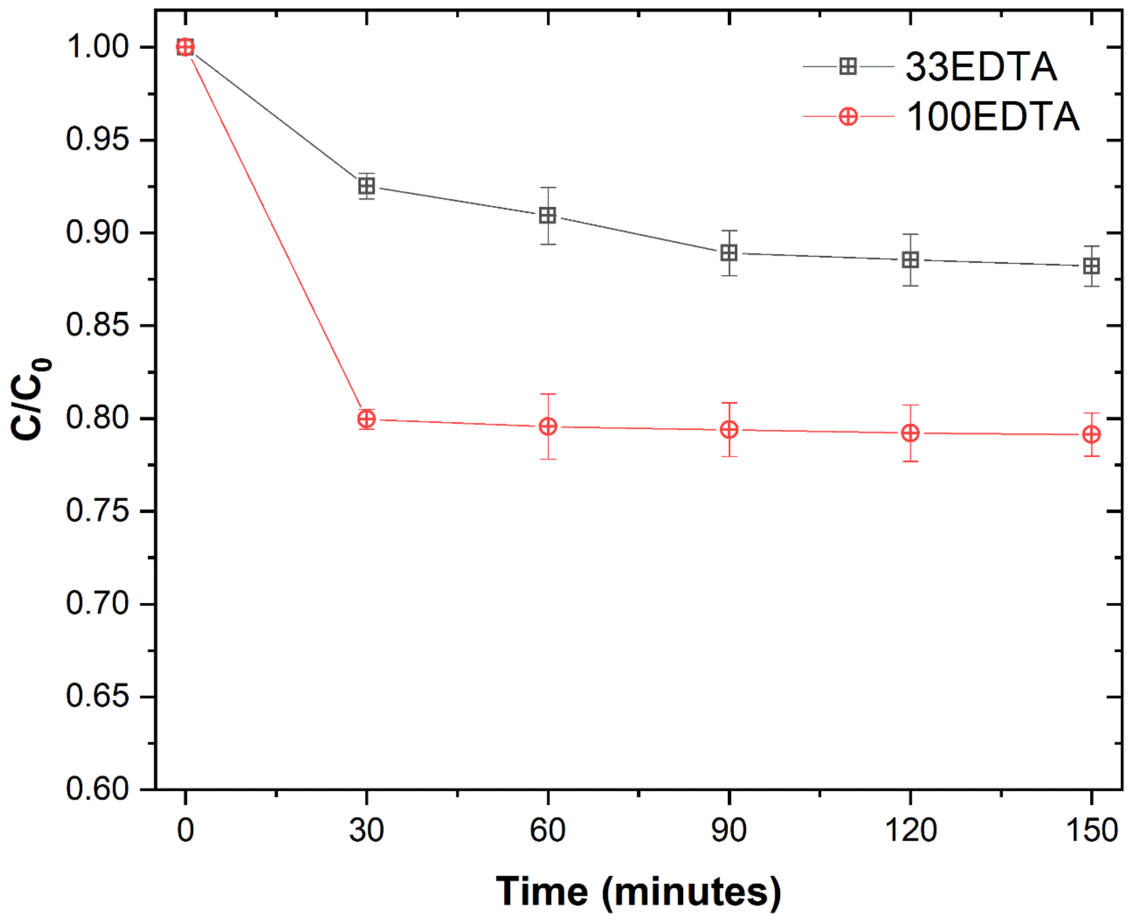

Figure S2. Adsorption tests performed for 150 minutes using methylene blue (MB) dye for the 33EDTA and 100EDTA samples.
